# Defining Metabolically Healthy Obesity: Role of Dietary and Lifestyle Factors

**DOI:** 10.1371/journal.pone.0076188

**Published:** 2013-10-17

**Authors:** Catherine M. Phillips, Christina Dillon, Janas M. Harrington, Vera J. C. McCarthy, Patricia M. Kearney, Anthony P. Fitzgerald, Ivan J. Perry

**Affiliations:** 1 Health Research Board Centre for Diet and Health Research, Department of Epidemiology and Public Health, University College Cork, Cork, Ireland; 2 Department of Statistics, University College Cork, Cork, Ireland; Postgraduate Medical Institute & Hull York Medical School, University of Hull, United Kingdom

## Abstract

**Background:**

There is a current lack of consensus on defining metabolically healthy obesity (MHO). Limited data on dietary and lifestyle factors and MHO exist. The aim of this study is to compare the prevalence, dietary factors and lifestyle behaviours of metabolically healthy and unhealthy obese and non-obese subjects according to different metabolic health criteria.

**Method:**

Cross-sectional sample of 1,008 men and 1,039 women aged 45-74 years participated in the study. Participants were classified as obese (BMI ≥30kg/m^2^) and non-obese (BMI <30kg/m^2^). Metabolic health status was defined using five existing MH definitions based on a range of cardiometabolic abnormalities. Dietary composition and quality, food pyramid servings, physical activity, alcohol and smoking behaviours were examined.

**Results:**

The prevalence of MHO varied considerably between definitions (2.2% to 11.9%), was higher among females and generally increased with age. Agreement between MHO classifications was poor. Among the obese, prevalence of MH was 6.8% to 36.6%. Among the non-obese, prevalence of metabolically unhealthy subjects was 21.8% to 87%. Calorie intake, dietary macronutrient composition, physical activity, alcohol and smoking behaviours were similar between the metabolically healthy and unhealthy regardless of BMI. Greater compliance with food pyramid recommendations and higher dietary quality were positively associated with metabolic health in obese (OR 1.45-1.53 unadjusted model) and non-obese subjects (OR 1.37-1.39 unadjusted model), respectively. Physical activity was associated with MHO defined by insulin resistance (OR 1.87, 95% CI 1.19-2.92, *p* = 0.006).

**Conclusion:**

A standard MHO definition is required. Moderate and high levels of physical activity and compliance with food pyramid recommendations increase the likelihood of MHO. Stratification of obese individuals based on their metabolic health phenotype may be important in ascertaining the appropriate therapeutic or intervention strategy.

## Introduction

Obesity prevalence is increasing worldwide, with the condition predicted to affect more than one billion people by 2030 [1]. Obesity is associated with increased risk of developing co-morbidities including type 2 diabetes mellitus (T2DM) and cardiovascular disease (CVD) [2,3], leading to increased risk of premature death. Consistent with this are recent data from a very large meta-analysis confirming significantly higher all-cause mortality with obesity when all grades are combined [4]. However grade 1 obesity (BMI 30 to < 35 kg/m^2^) was not associated with higher mortality. It is known that the obese phenotype may exist in the absence of metabolic abnormalities such as dyslipidaemia, insulin resistance and hypertension [5–7], which may partly explain these conflicting findings. Likewise not all non-obese individuals present a healthy metabolic and disease-free profile. There has been much interest in the paradoxical finding of individuals considered “metabolically healthy” despite increased adiposity. Subsequently several phenotype subgroups of obesity have been described including metabolically healthy obese (MHO) [6–11].

Determinants of MHO are unclear, particularly regards dietary and lifestyle behaviours [12,13]. Limited data on dietary composition and MHO exist. Results from a recent examination of dietary composition (food groups), macro- and micronutrient intakes and physical activity in a multi-ethnic group of 775 obese Americans do not support the hypothesis that dietary composition or physical activity are associated with MH [14]. Despite the knowledge of the MHO phenotype for some time now there are no unique criteria to define MH, resulting in widely varying prevalence estimates (6-35%) depending on which criteria are used [15,16]. No comparative data examining both dietary composition/quality and physical activity using a variety of MH definitions are currently available.

Current obesity treatment guidelines do not distinguish between MHO and metabolically unhealthy obesity (MUO), and recommend treatment for all obese individuals, starting with lifestyle intervention [17]. While studies examining the impact of dietary and exercise interventions in MHO are sparse and have produced conflicting results [18–21], they highlight the potential benefits of differentiating MHO and MUO individuals. Stratification of obese individuals based on their metabolic health phenotype may be important in determining the appropriate therapeutic strategy. The main objectives of this paper are to examine metabolic health prevalence in obese and non-obese men and women across a range of definitions and to comprehensively investigate to what extent differences between metabolically healthy and unhealthy obese and non-obese subjects are explained by dietary and lifestyle factors.

## Materials and Methods

### Study design and subject recruitment

The Cork and Kerry Diabetes and Heart Disease Study (Phase II) was a single centre, cross-sectional study conducted between 2010 and 2011 [22]. In brief, a population representative random sample was recruited from a large primary care centre in Mitchelstown, County Cork, Ireland (Mitchelstown cohort). The Livinghealth Clinic includes 8 general practitioners and serves a catchment area of approximately 20,000 with a mix of urban and rural residents. Participants were randomly selected from all registered attending patients in the 50-69 year age group. In total 3,807 potential participants were selected from the practice list. Following exclusion of duplicates, deaths and ineligibles, 3,043 were invited to participate in the study and of these 2,047 (49.2% male) completed the questionnaire and physical examination components of the assessment (response rate 67%). Ethics committee approval conforming to the Declaration of Helsinki was obtained from the Clinical Research Ethics Committee of University College Cork. All participants provided written informed consent. Following exclusion of missing BMI data the remaining 2,040 participants were analysed.

### Clinical data

Participants attended the clinic in the morning after an overnight fast (minimum 8h). Fasting blood samples were taken on arrival. Participants completed a General Health Questionnaire (GHQ), a Food Frequency Questionnaire (FFQ) and the International Physical Activity Questionnaire (IPAQ). Data on age, gender, family history, medication use (diabetes, dyslipidaemia and hypertension) and medical history (diabetes, hypertension and cardiovascular disease) was gathered through a self-completed GHQ. Blood pressure was measured according to the European Society of Hypertension Guidelines using an Omron M7 Digital BP monitor on the right arm, after a 5 minute rest in the seated position. The average of the second and third measurements was used for analyses.

### Anthropometric data

Anthropometric measurements were recorded with calibrated instruments according to a standardised protocol. Body weight was measured in kilograms without shoes, to the nearest 100g, using a Tanita WB100MA weighing scales (Tanita Corporation, IL, USA). Height was measured in centimetres to 1 decimal place using a Seca, Leicester height gauge (Seca, Birmingham, UK). Waist circumference (defined as mid-way between lowest rib and iliac crest) was measured in centimetres to 1 decimal place using a Seca 200 measuring tape (Seca, Birmingham, UK). The average of two measures were used for analyses. Body mass index (BMI) was calculated. Body fat percentage (BF%) was estimated using a recently described body adiposity estimator [23]. Individuals with a BMI ≥30kg/m^2^ were defined as obese.

### Dietary and lifestyle data

Diet was assessed using a modified version of the self-completed EPIC FFQ used in the Cork and Kerry Phase 1 study [24] which has been validated for use in the Irish population. A dietary score (the DASH (Dietary Approaches to Stop Hypertension) score) was calculated using the FFQ responses. It was a composite score derived from standard food groups within the FFQ as described by Fung et al. [25]. For each food group, consumption was divided into quintiles and participants were classified according to their intake ranking. Consumption of healthy food components were rated on a scale of 1-5, the higher the score the more frequent the consumption of that food. Less healthy dietary constituents, where low consumption is desired, were scored on a reverse scale with lower consumption receiving the higher scores. Component scores were summed and an overall DASH score for each person was calculated. A lower score indicated a poorer dietary quality. The DASH score was also categorised by median. Daily servings from each food pyramid shelf and compliance with the food pyramid were also determined.

Physical activity levels were assessed using the short form IPAQ [26] which provided information on frequency, duration and intensity of physical activity. Physical activity was categorized into 3 groups; low, moderate and high, based on a combination of activity frequency, duration of each activity bout and metabolic equivalent (MET) minutes per week in all activity types. Subjects were additionally classified according to current Irish physical activity guidelines of at least 30 minutes of moderate exercise 5 days a week or 150 minutes per week. Smoking status was defined as never, former and current smokers. Alcohol consumption included questions based on weekly intake to define never, moderate and heavy drinkers [27]. The Alcohol Use Disorders Identification Test (AUDIT-C) was used to identify subjects who are hazardous drinkers or have active alcohol use disorders.

### Biological analyses

Plasma and serum were prepared from fasting blood samples from each subject. Fasting plasma glucose (FPG), serum total, high density lipoprotein (HDL) cholesterol, low density lipoprotein (LDL) cholesterol and triglycerides levels were measured by Cork University Hospital Biochemistry Laboratory using fresh blood samples. FPG concentrations were determined using a glucose hexokinase assay and serum lipids were analysed using enzymatic colorimetric tests (Olympus Life and Material Science Europa Ltd., Lismeehan, Co. Clare, Ireland) on an Olympus 5400 automatic analyser (Olympus Diagnostica Gmbh, Hamburg, Germany). Serum insulin and C reactive protein (CRP) were determined using a biochip array system (Evidence Investigator; Randox Laboratories, UK). Homeostasis model assessment of insulin resistance (HOMA-IR) was derived from fasting glucose and insulin concentrations as [(fasting plasma glucose x fasting serum insulin)/ 22.5] [28].

### Determination of metabolic health status

Subjects were classified according to their BMI as obese (BMI ≥30kg/m^2^) and non-obese (BMI <30kg/m^2^). Metabolic health status was defined using a selection of five existing MH definitions based on a range of cardiometabolic abnormalities [9,29–32] (Table 1). In secondary analyses we also examined the combined overweight and obese (BMI ≥25kg/m^2^) subjects and the normal weight only (BMI <25kg/m^2^) subjects generating the following groups: metabolically healthy overweight and obese (MHOWO), metabolically unhealthy overweight and obese (MUOWO), metabolically healthy normal weight (MHN) and metabolically unhealthy normal weight (MUN).

**Table 1:** Criteria used to define metabolic health status.

	**Aguilar-Salinas**	**Karelis**	**Meigs (A)^1^**	**Meigs (B)^2^**	**Wildman**
Blood pressure	SBP <140 and DBP <90 or no treatment	-	SBP ≥130 or DBP ≥85 or treatment	-	SBP ≥130 or DBP ≥85 or treatment
TAG, mmol/L	-	≤1.70	≥1.70	-	≥1.70
HDL-C, mmol/L	≥1.04	≥1.30 and no treatment	<1.04 (M) <1.30 (F)	-	<1.04 (M) <1.30 (F) or treatment
LDL-C, mmol/L	-	≤2.60 and no treatment	-	-	-
Total-C, mmol/L	-	≥5.20	-	-	-
FPG, mmol/L	<7.00 and no treatment	-	≥5.60 or treatment	-	≥5.55 or treatment
HOMA		≤1.95		<75^th^ percentile^3^	>90^th^ percentile
Other	-	-	Waist >102cm (M)Waist >88cm (F)	-	CRP >90^th^ percentile
MH criteria	All of the above	≥4 of the above	<3 of the above	All of the above	<2 of the above
Study population	Mexico *n* = 716, 26.4% male	Canada *n* = 154, 0% male	US *n* = 2902, 45% male	US *n* = 2902, 45% male	US *n* = 5440, 47.9% male

Abbreviations: DBP: diastolic blood pressure; F: female; FPG: fasting plasma glucose; M: male; MH: metabolically healthy; SBP: systolic blood pressure. ^1^Using metabolic syndrome variables. ^2^Using homeostasis model only. ^3^Among non-diabetic subjects.

### Statistical analysis

Statistical analysis was conducted using PASW Statistics version 18.0 for Windows (SPSS Inc, Chicago, IL). Continuous variables are expressed as means ± SEM and categorical variables as percentages. Biochemical variables were assessed for normality of distribution, and skewed variables were normalised by log_10_ or square root transformation as appropriate. Differences between groups were analysed by independent *t*-tests for continuous variables and Chi-Square test for categorical variables. Logistic regression was used to determine associations between MHO and MHNO, demographic, dietary and lifestyle factors. Multivariate logistic regression analysis was performed including age, gender, dietary quality, food pyramid compliance, physical activity, smoking status and alcohol consumption as confounding factors. Pair-wise comparison of agreement between metabolic health definitions was assessed by Cohen’s kappa where values >0.75, 0.4-0.75 and <0.4 indicate excellent, fair to good and poor agreement, respectively [33]. A *P*-value of < 0.05 was considered significant.

## Results

### Prevalence of metabolically healthy and unhealthy obese and non-obese phenotypes

**Figure 1:**
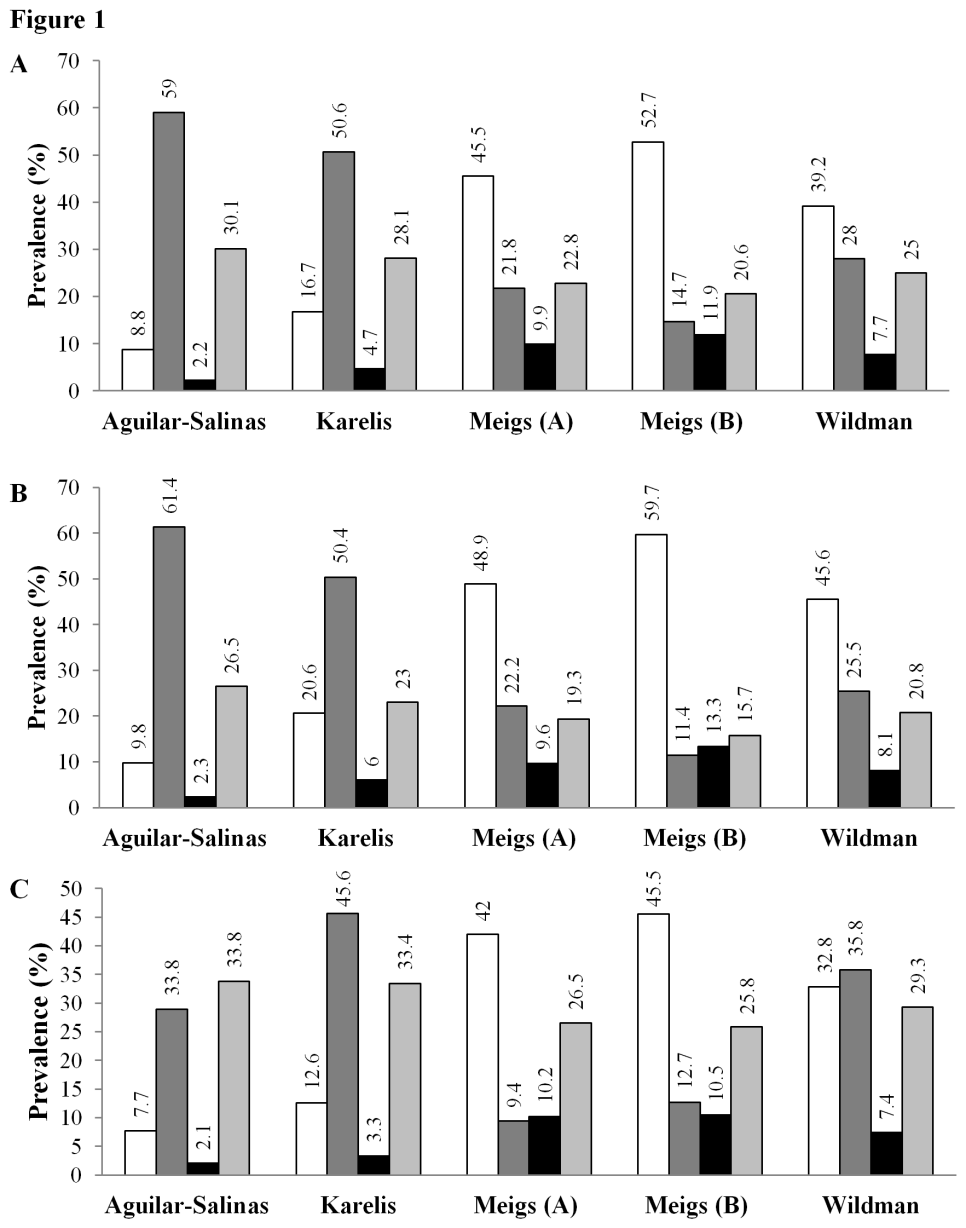
Prevalence of metabolically healthy and unhealthy obese and non-obese phenotypes in the Mitchelstown cohort according to different metabolic health criteria among all subjects and stratified by gender. Results are expressed as the percentage of all subjects (**A**), female (**B**) and male (**C**) participants within each metabolic health definition. The metabolically healthy non-obese (MHNO), metabolically unhealthy non-obese (MUNO), metabolically healthy obese (MHO) and metabolically unhealthy obese (MUO) groups are depicted as white, dark grey, black bars and light grey bars, respectively.

**Figure 2:**
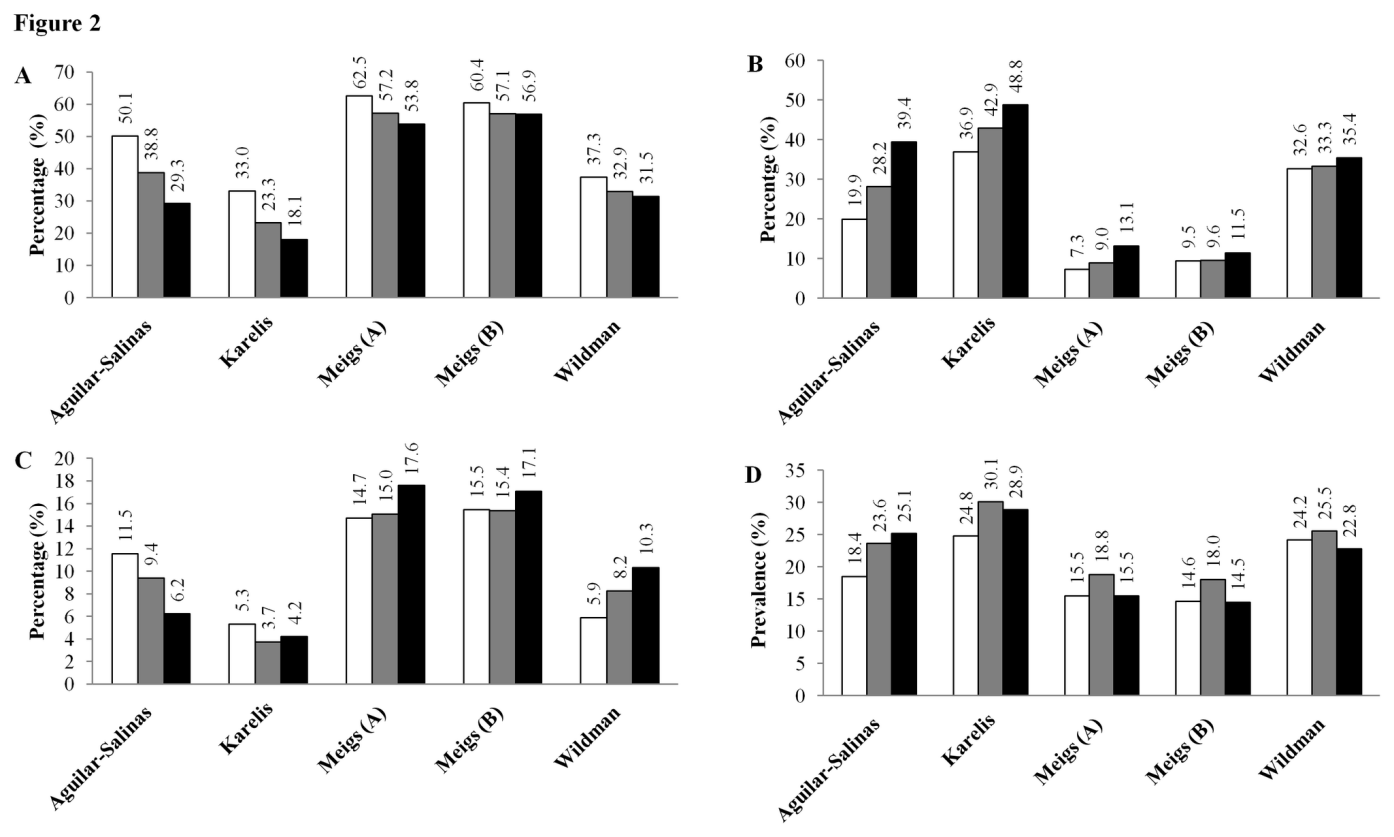
Prevalence of metabolically healthy and unhealthy obese and non-obese phenotypes according to different criteria stratified by age group. The prevalence of metabolically healthy non-obese subjects (**A**) decreased with age according to each definition. Conversely estimates of metabolically unhealthy non-obese subjects (**B**) increased with age according to each definition. Metabolically healthy obesity (**C**) prevalence differed according to which definition was used. The Aguilar-Salinas et al., and Karelis et al., definitions identified the highest prevalence among the 45-54 yr olds whereas the Wildman et al., and Meigs et al., definitions found the greatest prevalence of MHO among the 65-74 yr olds. Prevalence of metabolically unhealthy obesity (**D**) was consistently highest among the 55-64 yr olds, with the exception of the Aguilar-Salinas et al., definition. Meigs A is defined using metabolic syndrome variables and Meigs B is defined using HOMA. Results are expressed as percentage of all subjects within each age category. The 45-54, 55-64 and 65-74 yr old age groups are depicted as white, grey and black bars, respectively.

The proportion of individuals in the Mitchelstown cohort defined as being metabolically healthy by BMI categories varied considerably between definitions (Figure **1A**). Among all individuals, 2.2% to 11.9%, were obese yet metabolically healthy (MHO), 20.6% to 30.1% were obese and metabolically unhealthy (MUO), 14.7% to 59% were non-obese yet metabolically unhealthy (MUNO), and 8.8% to 52.7% were non-obese and metabolically healthy (MHNO). Among the obese participants, the proportion of metabolically healthy subjects ranged from 6.8% (Aguilar-Salinas), 14.2% (Karelis), 23.7% (Wildman), 30.2% (Meigs (A)) to 36.6% (Meigs B). Among the non-obese participants, the proportion of metabolically unhealthy subjects ranged from 21.8% (Meigs B), 32.4% (Meigs (A)), 41.7% (Wildman), 75.2% (Karelis) to 87% (Aguilar-Salinas). Examination of metabolic health obese phenotypes according to gender revealed higher prevalence of metabolic health among both obese and non-obese females, whereas the prevalence of the metabolically unhealthy phenotype was generally greater among obese and non-obese males (Figure 1B and 1C). MHO prevalence increased with age, except for MHO defined by Aguilar-Salinas and Karelis (Figure 2), whereas the prevalence of MHNO decreased with age according to each definition. Secondary analysis of the prevalence estimates of MHOWO, MUOWO, MHN and MUN followed the same definition dependent trends identified in the primary analysis (Table S1).

### Agreement between MHO classifications

**Figure 3:**
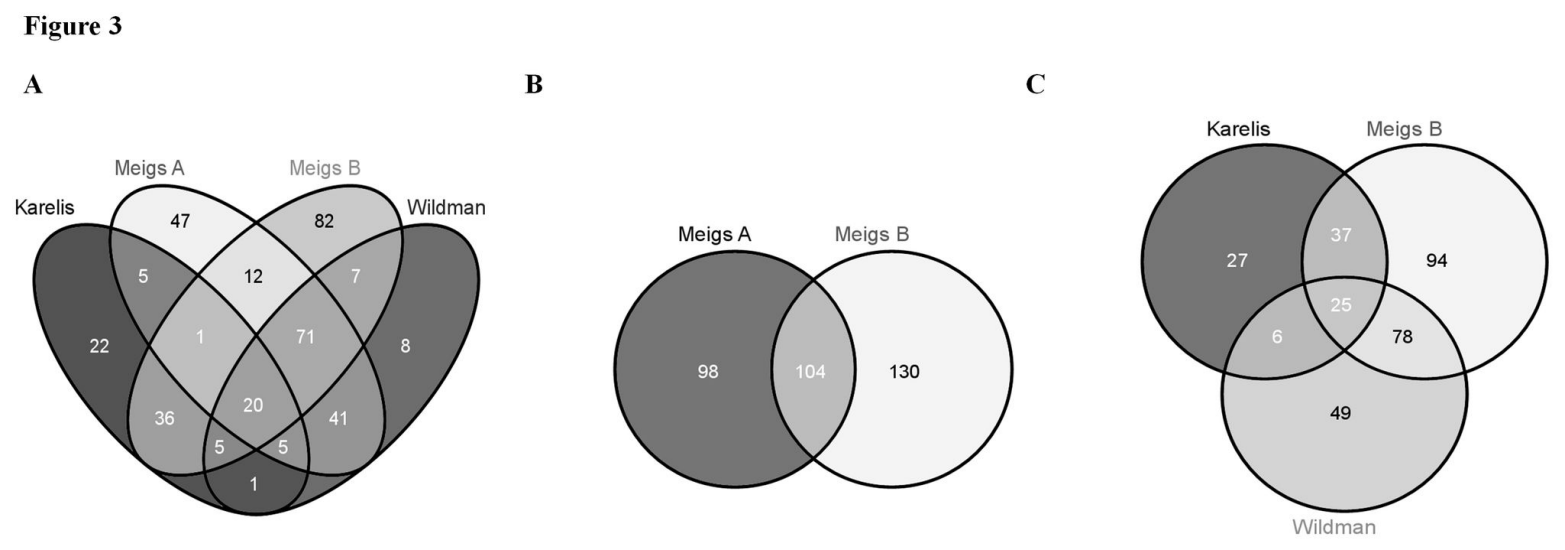
Agreement between MHO definitions. Only 20 subjects were simultaneously classified as MHO according to the four definitions which led to the greatest prevalence (Figure 3A). Less than half (44.4%) of the MHO subjects identified by Meigs B were simultaneously classified by Meigs A (Cohen’s kappa 0.23, *p* < 0.001) (Figure 3B). Comparison of the three MH definitions including HOMA identified only 25 subjects being similarly classified as MHO (Figure 3C).

We examined whether the same individuals were classified as MHO according to the different definitions. Comparison of the four definitions which led to the greatest prevalence (Karelis et al., Meigs et al., and Wildman et al.) revealed that only 20 subjects were simultaneously classified as MHO (Figure 3A). Examination of the Meigs et al., definitions revealed some degree of concordance. These scores identified a total of 436 MHO subjects: n=202 (Meigs A) and n=234 (Meigs B). Of the 234 participants classified as MHO according to Meigs B, 44.4% were simultaneously classified as MHO by Meigs A (Cohen’s kappa 0.23, *p* < 0.001) (Figure 3B). Comparison of the three MH definitions which included HOMA (Karelis et al., Meigs et al., (B) and Wildman et al.) identified only 25 subjects being similarly classified as MHO (Figure 3C).

### Anthropometric measures and clinical characteristics

Clinical and anthropometric characteristics of the study population according to both metabolic health status and BMI category are presented in Table 2. Compared to their metabolically unhealthy counterparts both the MHNO and MHO subjects had smaller waist circumference, lower BMI, TAG, FPG and higher HDL-C concentrations, reduced insulin resistance and were less hypertensive, with the exception of the MHO subjects defined by Aguilar-Salinas. In the secondary analyses comparison of the MHOWO and MUOWO subjects mirrored these findings, whereas some definition dependent differences were noted between the MHN and MUN subjects (Table S2).

**Table 2:** Anthropometric and clinical characteristics of the Mitchelstown cohort according to metabolic health by BMI category.

		**Aguilar-Salinas**	*P*	**Karelis**	*P*	**Meigs (A**)^1^	*P*	**Meigs (B**)^2^	*P*	**Wildman**	*P*
**Age, yrs**	MHO	58.9±0.8		61.5±0.5		59.3±0.4		60.1±0.3		59.9±0.5	
	MUO	59.9±0.2	0.21	59.9±0.2	0.007	60.4±0.2	0.01	59.9±0.3	0.74	60.1±0.2	0.79
	MHNO	59.7±0.4		60.4±0.3		58.7±0.2		59.4±0.2		58.5±0.2	
	MUNO	59.6±0.2	0.72	59.3±0.2	0.002	61.4±0.2	0.000	60.5±0.3	0.004	61.1±0.2	0.000
**BMI, kg/m^2^**	MHO	33.0±0.5		33.3±0.3		32.9±0.2		32.8±0.2		32.7±0.2	
	MUO	34.0±0.1	0.06	34.0±0.1	0.000	34.2±0.1	0.000	34.3±0.2	0.000	34.2±0.2	0.000
	MHNO	25.9±0.2		25.7±0.1		25.6±0.1		25.6±0.1		25.6±0.1	
	MUNO	26.1±0.1	0.003	26.2±0.1	0.000	27.0±0.1	0.000	27.6±0.1	0.000	26.8±0.1	0.000
**Waist, cm**	MHO	106.2±1.5		105.5±1.0		105.9±0.7		104.9±0.6		105.4±0.8	
	MUO	109.2±0.4	0.09	109.8±0.4	0.07	110.6±0.5	0.000	111.3±0.5	0.000	110.4±0.5	0.000
	MHNO	89.1±0.7		89.0±0.5		89.3±0.3		89.5±0.3		89.0±0.3	
	MUNO	91.3±0.3	0.29	91.8±0.3	0.004	94.9±0.5	0.000	96.6±0.5	0.000	93.9±0.4	0.000
**Body fat, %**	MHO	41.2±1.1		43.1±0.7		40.9±0.5		41.7±0.4		41.1±0.1	
	MUO	41.3±0.3	0.88	40.9±0.3	0.003	41.4±0.3	0.38	41.0±0.3	0.26	41.3±0.3	0.68
	MHNO	33.9±0.5		34.4±0.4		33.1±0.2		33.6±0.2		33.6±0.2	
	MUNO	33.7±0.2	0.70	33.5±0.2	0.02	34.9±0.3	0.000	34.1±0.4	0.22	33.9±0.3	0.39
**TG, mmol/L**	MHO	1.43±0.0		1.13±0.04		1.17±0.03		1.39±0.04		1.15±0.03	0.000
	MUO	1.65±0.03	0.03	1.74±0.04	0.000	1.86±0.05	0.000	1.79±0.05	0.000	1.79±0.04	0.000
	MHNO	1.20±0.03		0.96±0.02		1.13±0.02		1.17±0.02		1.06±0.02	
	MUNO	1.30±0.02	0.000	1.40±0.03	0.000	1.61±0.05	0.000	1.74±0.06	0.000	1.61±0.04	0.000
**HDL-C, mmol/L**	MHO	1.36±0.03		1.52±0.03		1.45±0.0		1.41±0.02		1.45±0.02	
	MUO	1.30±0.01	0.09	1.26±0.01	0.000	1.23±0.02	0.000	1.24±0.02	0.000	1.25±0.02	0.000
	MHNO	1.58±0.02		1.60±0.02		1.56±0.01		1.57±0.01		1.61±0.01	
	MUNO	1.52±0.01	0.03	1.50±0.01	0.000	1.43±0.02	0.000	1.33±0.02	0.000	1.40±0.02	0.000
**FPG, mmol/L**	MHO	5.20±0.09		5.27±0.13		4.96±0.03		5.01±0.04		4.87±0.03	
	MUO	5.53±0.06	0.006	5.58±0.07	0.03	5.78±0.08	0.000	5.87±0.09	0.000	5.73±0.07	0.000
	MHNO	4.86±0.05		4.92±0.04		4.85±0.02		4.85±0.02		4.78±0.02	
	MUNO	5.05±0.03	0.000	5.08±0.03	0.003	5.42±0.07	0.000	5.73±0.10	0.000	5.39±0.06	0.000
**HOMA**	MHO	3.71±0.65		2.81±0.37		3.02±0.17		1.66±0.04		2.21±0.13	
	MUO	4.65±0.19	0.17	4.93±0.20	0.000	5.33±0.24	0.000	6.33±0.25	0.000	5.35±0.22	0.000
	MHNO	1.74±0.09		1.57±0.08		1.60±0.03		1.34±0.02		1.41±0.03	
	MUNO	2.04±0.06	0.004	2.15±0.06	0.000	2.84±0.13	0.000	4.37±0.15	0.000	2.81±0.10	0.000
**SBP, mm Hg**	MHO	124.3±1.4		133.0±1.7		130.6±1.2		132.4±1.1		128.4±1.4	
	MUO	134.4±0.7	0.000	133.7±0.7	0.68	134.9±0.7	0.002	134.1±0.8	0.23	135.2±0.7	0.000
	MHNO	119.9±0.8		127.1±0.9		124.3±0.5		126.7±0.5		122.6±0.5	
	MUNO	128.8±0.5	0.000	127.9±0.5	0.43	134.5±0.8	0.000	132.2±0.9	0.000	134.8±0.7	0.000
**DBP, mm Hg**	MHO	78.1±1.1		80.5±0.9		81.7±0.7		82.1±0.6		80.3±0.8	
	MUO	82.9±0.4	0.000	81.8±0.4	0.04	82.8±0.4	0.19	82.7±0.5	0.43	83.2±0.4	0.002
	MHNO	75.2±0.5		77.4±0.5		77.6±0.3		78.4±0.3		76.8±0.3	
	MUNO	79.5±0.3	0.000	79.5±0.3	0.000	81.9±0.5	0.000	81.6±0.5	0.000	82.0±0.4	0.000

^1^Values are presented as means ± SEM. ^2^Statistical analysis conducted using Students *t*-tests. The first *p* value represents the metabolically healthy non-obese vs metabolically unhealthy non-obese comparison. The second *p* value represents the metabolically healthy obese vs metabolically unhealthy obese comparison.

### Dietary composition, quality and food pyramid shelf servings

No differences in calorie consumption, macronutrient intake and dietary quality were observed between MHO and MUO subjects (Table 3), with the exception of MHO subjects defined by Meigs A who had higher fat and lower carbohydrate intakes. Some definition dependent differences were noted between daily servings of fruit and vegetables, dairy, meats and fats between MHO and MUO subjects. Compliance with the food pyramid recommendations was greater among the MHO subjects (Meigs A and Wildman). Among the non-obese subjects (Table 4), calorie intake and dietary macronutrient composition were similar, with the exception of MHNO defined by Aguilar-Salinas. Dietary quality was higher among the MHNO subjects defined by Karelis and Meigs B. Daily number of servings of fruit and vegetables and dairy were higher among the MHNO defined by Meigs A, Meigs B and Wildman. Micronutrient intakes were examined. MHO subjects had lower retinol intake (Aguilar-Salinas and Meigs B) and higher iodine intake (Meigs A and Wildman) compared to the MUO subjects (*p* < 0.05, data not shown). The secondary analyses did not reveal any significant differences regards energy intake, macronutrient composition and dietary quality between the MHOWO and MUOWO subjects. Comparison of the MHN and MUN subjects produced similar findings to comparison between MHNO and MUNO subjects (data not shown).

**Table 3:** Dietary and lifestyle factors according to metabolic health status among the obese Mitchelstown cohort participants.

	**Aguilar-Salinas**		**Karelis**		**Meigs (A)^1^**			**Meigs (B)^2^**		**Wildman**	
	MHO	MUO	MHO	MUO	MHO		MUO	MHO	MUO	MHO	MUO
	*n* = 42	*n* = 574	*n* = 95	*n* = 573	*n* = 202		*n* = 466	*n* = 234	*n* = 405	*n* = 158	*n* = 510
**Dietary composition and quality**											
Kilocalories	2224±172	2039±32	2152±97	2042±33	1970±52		2096±39	2059±55	2060±40	1984±59	2080±37
Fat (% EI)	34.5±0.9	34.1±0.3	34.5±0.7	34.0±0.3	34.9±0.5*		33.7±0.2	34.1±0.5	34.1±0.3	35.0±0.6	33.8±0.3
Carbohydrate (% EI)	47.7±1.4	48.8±0.3	48.2±0.9	48.8±0.3	47.6±0.6*		49.1±0.4	48.6±0.6	48.9±0.4	47.8±0.7	48.9±0.4
Protein (% EI)	19.6±0.6	18.5±0.2	18.3±0.4	18.6±0.2	18.6±0.3		18.6±0.3	18.2±0.3	18.8±0.2	18.4±0.3	18.6±0.2
Fibre (% EI)	2.57±0.11	2.58±0.03	2.52±0.07	2.56±0.03	2.46±0.06		2.60±0.03	2.55±0.05	2.57±0.04	2.48±0.06	2.58±0.03
Sugar (% EI)	19.5±1.3	20.6±0.3	19.8±0.9	20.7±0.3	20.5±0.6		20.6±0.3	20.8±0.5	20.5±0.4	20.5±0.6	20.6±0.3
Dietary quality	26.9±1.3	28.5±0.3	29.4±0.7	28.3±0.3	28.5±0.3		28.4±0.3	28.4±0.4	28.4±0.3	28.4±0.5	28.4±0.3
**Daily food pyramid shelf servings**											
Bread, cereal, potatoes, grain and rice	4.6±0.4	5.2±0.1	5.5±0.4	5.1±0.1	5.3±0.2	5.1±0.1		5.4±0.2	5.0±0.1	5.5±0.2	5.0±0.1
Fruit and vegetable	5.3±0.6**	7.2±0.2	6.8±0.4	7.0±0.2	7.7±0.4*	6.7±0.2		7.3±0.4	6.8±0.2	7.6±0.4	6.8±0.2
Dairy	1.8±0.2	1.9±0.1	2.3±0.2*	1.9±0.1	2.0±0.1	1.9±0.1		1.9±0.1	1.9±0.1	2.0±0.1	1.9±0.1
Meat, fish, poultry, eggs	2.0±0.2**	2.5±0.1	2.5±0.2	2.5±0.1	2.5±0.1	2.5±0.1		2.5±0.1	2.5±0.1	2.6±0.1	2.4±0.1
Fats and high fat/sugar foods and drinks	8.3±0.9	7.9±0.2	7.8±0.6	8.0±0.2	8.5±0.4	7.7±0.2		8.0±0.4	7.9±0.3	8.8±0.5*	7.7±0.2
Food pyramid compliance	2.6±0.2	2.4±0.1	2.5±0.1	2.4±0.1	2.5±0.1*	2.3±0.1		2.5±0.1	2.3±0.1	2.5±0.01*	2.4±0.1
**Physical activity (%)**											
Low activity	54.2	61.5	55.2	48.9	55.4	51.9		59.1	47.7	55.3	51.0
Moderate activity	28.6	23.1	26.8	35.9	27.9	28.6		25.6	31.4	27.3	30.9
High activity	17.2	15.4	18.0	15.2	16.7	19.6		15.3	20.9	17.4	18.1
Meeting activity recommendations	6.1	19.0	17.7	18.8	18.3	18.8		15.0	20.3	14.9	19.8
Total PA (mins/day)^3^	83±25.6	117±9.1	111±20.6	119±9.3	115±13.1	119±10.8		126±13.0	113±12.0	108±14.2	121 ±10.2
**Smoking status (%)**											
Never smoker	48.8	49.9	46.2	49.6	51.0	48.3		50.7	48.6	47.7	49.6
Former smoker	34.1	40.5	45.1	39.9	41.8	40.1		40.3	40.2	47.1*	38.6
Current smoker	17.1	9.6	8.7	10.5	7.1	11.6		9.0	11.2	5.2	11.8
**Alcohol intake (%)**											
Non-drinker	13.6	24.3	24.4	24.4	20.9	24.2		24.1	23.0	22.8	23.3
Moderate drinker	68.2	61.9	67.2	61.5	65.9	60.7		62.1	61.9	66.3	61.0
Heavy drinker	18.2	13.8	16.4	14.2	13.2	15.1		13.8	15.1	10.9	15.7
Hazardous drinker	77.8	71.0	75.0	71.1	67.9	73.3		67.7	74.7	62.1*	74.6
Within recommendations	90.9	93.2	90.2	93.2	94.6	91.9		91 .0	93.3	93.1	92.7

^1^Using metabolic syndrome variables. ^2^Using homeostasis model only. ^3^Daily physical activity duration based on a typical day of activity. Abbreviations: PA Physical activity. Values for continuous variables are presented as means ± SEM and categorical variables are presented as percentages. Statistical analysis conducted using Students *t*-test for continuous variables and Chi-Square test for categorical variables. * represents *P* < 0.05 compared to MUO obtained using *t*-tests. ** represents *P* < 0.01 compared to MUO obtained using *t*-tests.

**Table 4:** Dietary and lifestyle factors according to metabolic health status among the non-obese Mitchelstown cohort participants.

	**Aguilar-Salinas**		**Karelis**		**Meigs (A)^1^**			**Meigs (B)^2^**		**Wildman**		
	MHNO	MUNO	MHNO	MUNO	MHNO	MUNO		MHNO	MUNO	MHNO		MUNO
	*n* = 168	*n* = 1126	*n* = 340	*n* = 1032	*n* = 928	*n* = 444		*n* = 1036	*n* = 289	*n* = 800		*n* = 572
**Dietary composition and quality**												
Kilocalories	2220±73 *	2000±24	2092±44	2000±26	2015±28	2035±38		2042±26	1974±46	2030±31		2010±33
Fat (% EI)	32.6±0.6 **	33.8±0.2	34.0±0.4	33.4±0.2	33.5±0.2	33.6±0.2		33.7±0.2	33.2±0.4	33.7±0.3		33.4±0.3
Carbohydrate (% EI)	51.5±0.7 *	48.7±0.3	49.0±0.5	49.2±0.3	49.3±0.3	48.8±0.4		49.0±0.3	49.7±0.5	49.2±0.3		49.1±0.4
Protein (% EI)	17.9±0.3 **	18.6±0.1	18.6±0.2	18.5±0.1	18.6±0.1	18.5±0.1		18.6±0.1	18.2±0.2	18.5±0.2		18.6±0.4
Fibre (% EI)	2.74±0.6	2.59±0.02	2.57±0.4	2.62±0.02	2.62±0.02	2.58±0.04		2.60±0.02	2.65±0.05	2.61±0.03		2.61±0.03
Sugar (% EI)	22.2±0.7 *	20.5±0.2	20.6±0.4	20.9±0.3	20.9±0.3	20.4±0.4		20.7±0.2	21.2±0.4	20.9±0.3		20.7±0.3
Dietary quality^3^	28.9±0.6	29.1±0.2	29.8±0.4 **	28.8±0.2	29.1±0.2	29.1±0.3		29.2±0.2 **	28.2±0.4	29.2±0.2		28.8±0.3
**Daily food pyramid shelf servings**												
Bread, cereal, potatoes, grain and rice	5.3±0.3	5.3±0.1	5.3±0.2	5.3±0.1	5.4±0.1		5.1±0.1	5.3±0.1	5.2±0.2		5.4±0.1	5.2±0.1
Fruit and vegetable	7.5±0.6	7.2±0.1	7.5±0.3	7.1±0.2	7.4±0.2		6.9±0.2	7.4±0.2 *	6.5±0.3		7.6±0.2 *	6.7±0.2
Dairy	1.9±0.1	2.0±0.1	2.0±0.1	1.9±0.1	2.0±0.1 *		1.8±0.1	2.0±0.1 *	1.7±0.1		2.1±0.1 *	1.8±0.1
Meat, fish, poultry, eggs	2.4±0.1	2.4±0.1	2.3±0.1	2.4±0.1	2.4±0.1		2.3±0.1	2.4±0.1	2.4±0.1		2.4±0.1	2.3±0.1
Fats and high fat/sugar foods and drinks	7.5±0.4	7.9±0.2	7.6±0.3	8.0±0.2	8.0±0.2		7.6±0.2	7.9±0.2	7.8±0.3		8.0±0.2	7.7±0.2
Food pyramid compliance	2.4±0.1	2.4±0.1	2.4±0.1	2.3±0.1	2.3±0.1		2.4±0.1	2.4±0.1	2.3±0.1		2.3±0.1	2.4±0.1
**Physical activity (%)**												
Low activity	45.8	43.6	46.4	43.8	47.8		44.7	48.5	45.0		49.2	43.3
Moderate activity	30.3	31.4	30.6	29.0	29.3		30.6	32.1	30.0		25.7	33.4
High activity	23.9	25.0	23.0	27.2	22.9		24.6	19.4	25.0		25.1	23.3
Meeting activity recommendations	21.7	19.9	19.0	20.5	22.2		16.0	20.1	19.8		21.1	18.8
Total PA (mins/day)^3^	158±20.6	143±7.4	141±11.8	146±8.1	157±9.0**		121±9.3	143±7.4	156±12.7		149±0.0	140 ±10.0
**Smoking status (%)**												
Never smoker	61.3	51.3	56.7	51.0	53.7		49.8	53.1	51.6		54.3	49.9
Former smoker	24.5	31.6	27.3	31.8	29.7		32.7	29.6	34.5		29.1	32.8
Current smoker	14.1	17.1	16.1	17.2	16.6		17.5	17.1	13.9		16.6	17.3
**Alcohol intake (%)**												
Non-drinker	14.3	19.6	20.6	18.1	19.2		17.6	18.3	19.7		18.8	18.6
Moderate drinker	76.2	65.3	66.1	66.5	66.4		66.4	67.2	64.1		68.3	63.8
Heavy drinker	9.5	15.1	13.3	15.4	14.1		15.9	14.5	16.2		12.9	17.6
Hazardous drinker	67.5	69.9	65.1	71.0	69.2		70.4	69.2	72.4		68.5	71.0
Within recommendations	90.5	90.3	89.9	89.9	90.0		89.5	90.3	88.9		90.5	89.0

^1^Using metabolic syndrome variables. ^2^Using homeostasis model only. ^3^Daily physical activity duration based on a typical day of activity. Abbreviations: PA Physical activity. Values for continuous variables are presented as means ± SEM and categorical variables are presented as percentages. Statistical analysis conducted using Students *t*-test for continuous variables and Chi-Square test for categorical variables. * represents *P* < 0.05 compared to MUNO obtained using *t*-tests. ** represents *P* < 0.01 compared to MUNO obtained using *t*-test.

### Lifestyle behaviours: physical activity, smoking status and alcohol consumption

Examination of lifestyle behaviours among the obese subjects (Table 3) did not reveal any differences in level of physical activity intensity, daily total time of physical activity and whether subjects met current physical activity guidelines. Differences in smoking status and alcohol consumption were noted for MHO defined by Wildman, wherein fewer MHO subjects were current smokers and engaged in hazardous drinking. Among the non-obese subjects (Table 4), duration of physical activity was higher among the MHNO subjects defined by Meigs A. Physical activity level, smoking status and alcohol consumption were not different between the MHNO and MUNO subjects. In the secondary analyses examination of physical activity measures, smoking behaviour and alcohol consumption did not reveal any significant differences between the metabolically healthy and unhealthy subjects, regardless of BMI (data not shown).

### Determinants of the metabolically healthy phenotype

Among both the obese and non-obese subjects, females were more approximately 2-4 times more likely to be metabolically healthy relative to males (Table 5). Associations with age were conflicting. Among the obese subjects, greater compliance with food pyramid recommendations was positively associated with MH defined by Meigs A (OR 1.49, 95% CI 1.07-2.08, *p* = 0.018), Meigs B (OR 1.45, 95% CI 1.05-2.00, *p* = 0.025) and Wildman (OR 1.53, 95% CI 1.07-2.19, *p* = 0.021) in the unadjusted analyses only. In the adjusted analyses a moderate to high level of physical activity was positively associated with MHO defined by insulin resistance, particularly among those with a high level of activity (OR 2.35, 95% CI 1.28-4.34, *p* = 0.006) relative to those with a low level of physical activity. Odds of presenting with MHO defined by Karelis were greater among alcohol drinkers.

**Table 5:** Multivariate-adjusted odds ratios for the metabolically healthy phenotype associated with demographic and lifestyle factors among obese and non-obese individuals.

	**Aguilar-Salinas**		**Karelis**		**Meigs (A)^1^**		**Meigs (B)^2^**		**Wildman**	
**Gender**		*P*		*P*		*P*		*P*		*P*
Male	1 [reference]		1 [reference]		1 [reference]		1 [reference]		1 [reference]	
Female (obese)	0.60 (0.18-2.10)	0.43	3.85 (1.87-7.95)	**0.000**	2.02 (1.20-3.41)	**0.008**	2.69 (1.57-4.60)	**0.000**	2.99 (1.68-5.32)	**0.000**
Female (non-obese)	1.15 (0.68-1.94)	0.59	1.94 (1.32-2.84)	**0.001**	1.33 (0.94-1.90)	0.11	2.16 (1.45-3.23)	**0.000**	2.02 (1.44-2.84)	**0.000**
**Age group**										
45-54	1 [reference]		1 [reference]		1 [reference]		1 [reference]		1 [reference]	
55-64 (obese)	0.59 (0.20-1.84)	0.38	3.15 (1.20-8.30)	**0.02**	0.60 (0.34-1.08)	0.08	0.50 (0.28-0.90)	**0.02**	0.76 (0.40-1.42)	0.39
65-74 (obese)	0.32 (0.04-3.20)	0.30	4.80 (1.56-14.80)	**0.006**	0.59 (0.27-1.29)	0.18	1.30 (0.60-2.81)	0.50	0.85 (0.37-1.98)	0.71
55-64 (non-obese)	1.35 (0.76-2.40)	0.31	1.11 (0.73-1.70)	0.61	0.54 (0.36-0.81)	**0.003**	0.82 (0.53-1.27)	0.82	0.51 (0.35-0.75)	**0.001**
65-74 (non-obese)	0.88 (0.37-2.12)	0.78	1.82 (1.05-3.10)	**0.03**	0.25 (0.15-0.41)	**0.000**	0.70 (0.40-1.25)	0.70	0.28 (0.17-0.47)	**0.000**
**Dietary quality^3^**										
Low	1 [reference]		1 [reference]		1 [reference]		1 [reference]		1 [reference]	
High (obese)	0.76 (0.22-2.66)	0.67	1.41 (0.70-2.86)	0.34	1.21 (0.71-2.04)	0.48	1.21 (0.70-2.07)	0.50	1.00 (0.56-1.78)	0.99
High (non-obese)	0.79 (0.47-1.33)	0.38	1.17 (0.80-1.70)	0.43	0.97 (0.68-1.37)	0.85	1.13 (0.76-1.67)	0.55	0.93 (0.67-1.30)	0.67
**Food pyramid compliance^4^**										
Low	1 [reference]		1 [reference]		1 [reference]		1 [reference]		1 [reference]	
High (obese)	1.04 (0.33-3.20)	0.94	0.83 (0.42-1.65)	0.59	0.99 (0.60-1.65)	0.97	0.96 (0.57-1.62)	0.88	1.05 (0.60-1.83)	0.85
High (non-obese)	0.95 (0.57-1.57)	0.83	1.10 (0.77-1.59)	0.59	0.79 (0.49-1.37)	0.40	0.87 (0.59-1.27)	0.47	0.76 (0.55-1.05)	0.09
**Physical activity**										
Low	1 [reference]		1 [reference]		1 [reference]		1 [reference]		1 [reference]	
Moderate + High (obese)	0.87 (0.28-2.72)	0.80	1.09 (0.55-2.16)	0.81	1.23 (0.73-2.04)	0.44	1.77 (1.19-3.01)	**0.002**	1.34 (0.76-2.34)	0.30
Moderate + High (non-obese)	0.69 (0.42-1.15)	0.16	1.30 (0.89-1.89)	0.17	1.02 (0.91-1.44)	0.91	1.31 (0.89-1.93)	0.16	1.24 (0.89-1.73)	0.19
**Smoking**										
Never + Former	1 [reference]		1 [reference]		1 [reference]		1 [reference]		1 [reference]	
Current (obese)	2.17 (0.54-8.72)	0.28	0.74 (0.20-2.71)	0.65	0.42 (0.16-1.08)	0.07	0.89 (0.38-2.08)	0.80	0.36 (0.12-1.16)	0.07
Current (non-obese)	0.77 (0.46-1.77)	0.90	1.07 (0.66-1.74)	0.77	0.95 (0.61-1.49)	0.83	1.71 (0.97-3.00)	0.06	0.89 (0.58-1.37)	0.59
**Alcohol intake**										
Non-drinker	1 [reference]		1 [reference]		1 [reference]		1 [reference]		1 [reference]	
Drinker (obese)	1.48 (0.41-5.34)	0.55	2.81 (1.12-7.02)	**0.027**	1.49 (0.79-2.81)	0.21	1.53 (0.81-2.89)	0.19	1.51 (0.75-2.99)	0.24
Drinker (non-obese)	1.55 (0.76-3.13)	0.22	0.96 (0.61-1.50)	0.85	1.10 (0.73-1.68)	0.64	1.19 (0.75-1.89)	0.47	1.27 (0.85-1.89)	0.25

^1^Using metabolic syndrome variables. ^2^Using homeostasis model only. ^3^Dietary quality determined by DASH score. ^4^Median food pyramid compliance score. Figures are expressed as OR (95%CI). Reference group is metabolically unhealthy within same BMI category. Each factor is adjusted for every other factor in the table.

Among the non-obese subjects, again in the unadjusted analyses higher dietary quality was positively associated with MH defined by Karelis (OR 1.39, 95% CI 1.04-1.84, *p* = 0.024) and Meigs B (OR 1.37, 95% CI 1.00-1.87, *p* = 0.048). These differences did not remain significant in the multivariate adjusted analysis. No relationship was identified with tobacco smoking or alcohol consumption. While physical activity was positively associated with metabolic health defined by insulin resistance and by Wildman, these did not reach statistical significance. In the secondary adjusted analysis comparison of the MHOWO and MUOWO subjects mirrored these findings, particularly with regard to the positive associations between being female (for most definitions) and having a moderate to high level of physical activity with metabolic health defined by insulin resistance among the overweight and obese subjects (Table S3).

## Discussion

In this study we demonstrated considerable variation in the prevalence of MHO (2.2% to 11.9%), MHNO (8.8% to 52.7%), MUO (20.6% to 30.1%) and MUNO (14.7% to 59%). Agreement between MHO classifications was poor. Among the obese participants, the proportion of metabolically healthy subjects ranged from 6.8% by Aguilar-Salinas, 14.2% by Karelis, 23.7% by Wildman, 30.2% by Meigs (A) to 36.6% by Meigs B. Two recent studies examined the prevalence of MHO using a range of definitions [16,34]. Velho et al., reported MHO prevalence among male and female obese subjects of 25.1-35.3% by Aguilar-Salinas, 3.3-11.4% by Karelis, 15.8-21.9% by Wildman, 30.1-39.0% by Meigs (A) and 32.1-43.3% by Meigs B [16]. The Korean study of 186 male MHO subjects reported MHO prevalence among obese subjects of 24.2% by Meigs B, 28.5% by Karelis and 59.7% by Wildman [34]. Despite study design and population differences, all three comparative studies indicate that MHO prevalence differs considerably depending on which definition is used, making comparisons between studies difficult.

Prevalence of metabolic health was higher among both obese and non-obese females, in keeping with Velho et al. [16]. Increasing age was associated with higher prevalence of MHO defined by Karelis, Meigs A and Wildman, whereas prevalence of MHO defined by Aguilar-Salinas and Meigs B decreased with increasing age. Velho et al. [16], reported decreased MHO prevalence with age in both genders, whereas Yoo et al. [34], did not observe any age related differences, probably due to the younger and narrow age range of study participants (mean age 37 years). Whether MHO transitions to MUO over time is unclear, but it may account for the observed decreasing MHO prevalence with age. Regards metabolic characteristics, the MHO and MHNO subjects had lower BMI, smaller waist circumference, reduced insulin resistance, lower glucose concentrations, more favourable lipid profiles and blood pressure compared to their metabolically unhealthy counterparts. Interestingly estimated body fat percentage was not generally different between the MHO and MHNO subjects and their metabolically unhealthy counterparts.

Dietary and lifestyle factors play an important role in the development of insulin resistance, obesity, metabolic syndrome and T2DM [35,36]. Increased consumption of high-energy, high fat diets and deterioration in dietary quality coupled with increased sedentary behaviour, result in increased accumulation of adipose tissue and progression to overt obesity, which is associated with insulin resistance, low-grade inflammation and increased risk of associated cardiometabolic abnormalities [37]. The exact molecular mechanisms underlying the loss of optimal adipose functionality and insulin resistance observed with increasing obesity are unclear. No comparative data examining dietary composition, dietary quality and lifestyle behaviours using a range of MH definitions in male and female obese and non-obese adults exist. In our study total calorie intake and dietary macronutrient composition were similar between the metabolically healthy and unhealthy regardless of BMI, with the exception of MHO subjects defined by Meigs A who consumed more fat and less carbohydrate than the MUO subjects. Data from two American studies do not support the hypothesis that total energy or dietary macronutrient intakes are associated with MHO [14,38]. The Korean NHANES III analysed dietary patterns in metabolically obese normal weight (MONW) and reported that high carbohydrate intake, particularly of high carbohydrate snacks, was associated with higher prevalence of MONW in women, whereas a high protein diet reduced risk of MONW [39]. Unfortunately this study did not examine obese subjects. In the present work dietary quality was not different between MHO and MUO subjects, but was higher among MHNO subjects defined by Karelis and Meigs B, and was positively associated with MHNO in the unadjusted analyses. Better compliance with food pyramid recommendations observed among the MHO subjects (defined by HOMA and Wildman) was positively associated with MHO in the unadjusted logistic analyses only. Some differences were noted between number of daily servings of fruit and vegetables, dairy, meats, fats and high fat/sugar food and drinks between MHO and MUO subjects, depending on which criteria are used. Only one earlier study examined food groups in MHO and did not report any differences [14]. The Finnish Type 2 Diabetes (FIN D2D) survey examined fruit and vegetable intake and did not identify differences between individuals with or without the metabolic syndrome according to BMI category [40]. The diversity of our results highlights the need for consensus regarding MHO definition in order to develop targeted dietary approaches.

Physical activity is associated with health-related benefits leading to lower risk of obesity, CVD and T2DM [41–43]. In our study the level of physical activity intensity, daily total time of physical activity and proportion of subjects meeting current Irish physical activity guidelines were not different between MHO and MUO subjects. The FIN D2D survey reported similar amount of leisure time physical activity between obese men and women with or without the metabolic syndrome [40]. Yoo et al. [34], reported that male MHO subjects defined by HOMA (Meigs B) were more regular exercisers, consistent with Velho et al. [16]. We demonstrated that a moderate to high level of physical activity was positively associated with MHO and also MHOWO defined by HOMA. Leisure time physical activity was also associated with MHO in the NHANES 1999-2004 cohort [7], a finding that was replicated by Velho et al. [16]. Collectively these data suggest that the beneficial effects of physical activity on cardiometabolic risk factors are evident even among overweight and obese subjects. No differences in smoking status and alcohol consumption were observed between the MHNO and MUNO subjects, whereas fewer MHO subjects defined by Wildman were current smokers. Previous studies have produced conflicting results regards metabolic health and smoking [16,44] and alcohol consumption [7,16]. In the adjusted analyses alcohol drinkers had increased odds for presenting with MHO defined by Karelis. The beneficial effects of moderate alcohol consumption on HDL-C are well known, but detrimental effects of alcohol include raised triglyceride concentrations, insulin resistance and abdominal obesity, which may partly account for the lack of a relationship between alcohol and MHO.

Our study has several strengths including a high participation rate (67%) and inclusion of questionnaires to assess dietary and lifestyle behaviours. Notwithstanding these strengths some limitations can be identified. The cross-sectional study design limited our ability to make an inference about the causal relationships between dietary and lifestyle factors and MHO. The use of self-reported questionnaires are subject to potential inaccuracies, recall and reporting biases. BMI was used to classify subjects as obese. As BMI does not discriminate between lean and fat body mass, persons of short stature or muscular build may be misclassified. Future work may benefit from direct measurement of body fat using dual-energy X-ray absorptiometry (DEXA). Prospective studies tracking the development of cardiometabolic disease and mortality in MHO are required. The planned longitudinal follow-up of the Mitchelstown cohort will allow differences in disease incidences, transition from MHO to MUO, and all-cause mortality risk according to the different MH criteria to be ascertained.

Our findings could have important clinical and public health nutrition significance. There is a clear need to improve obesity diagnosis, particularly of obese subjects at greatest cardiometabolic risk, and to develop new preventative and therapeutic strategies and evidence based public health measures to attenuate disease development and reduce the significant economic cost of obesity. We have shown that assessing body fat percentage (BF%) and BMI to classify obesity may help identify individuals at greater cardiometabolic risk than BMI alone [45]. Those identified as obese using both tools had a more metabolically unhealthy profile and were not responsive to dietary intervention. Furthermore we have recently demonstrated that favourable inflammatory status is positively associated with metabolic health in obese and non-obese individuals [46]. Taken collectively these findings suggest that coordination of the pathways involved in nutrient handling, insulin signalling, inflammation and lipid metabolism is less disturbed in MHO and that the MUO subjects are simply metabolically overburdened, and thus no longer dietary responsive. Therefore stratification of obese individuals based on their metabolic health phenotype may be important not only in identification of individuals at greatest risk but also in ascertaining the appropriate therapeutic or intervention strategy.

In conclusion, our data suggest that physical activity and compliance with food pyramid recommendations increase the likelihood of MHO. The wide variation in the estimates of MHO and MUNO observed in the current work underscore the need for a single standard definition.

## Supporting Information

Table S1 **Prevalence of metabolic health status among the normal weight and combined overweight and obese subjects.**
(DOCX)

Table S2 **Anthropometric and clinical characteristics of the Mitchelstown cohort according to metabolic health status among the normal weight and combined overweight and obese subjects.**
(DOCX)

Table S3 **Multivariate-adjusted odds ratios for the metabolically healthy phenotype associated with demographic and lifestyle factors among the normal weight and the combined overweight and obese individuals.**
(DOCX)
