# Supplementary material for: Defining Metabolically Healthy Obesity: Role of Dietary and Lifestyle Factors
Source: PLoS One. 2013 Oct 17;8(10):e76188. doi: 10.1371/journal.pone.0076188 (PMC3798285; doi:10.1371/journal.pone.0076188)
Supplement: Table S1 — Prevalence of metabolic health status among the normal weight and combined overweight and obese subjects. (DOCX) [file pone.0076188.s001.docx]

**Table S1.** Prevalence of metabolic health status among the normal weight and combined overweight and obese subjects

|  | **Aguilar-Salinas** | **Karelis** | **Meigs (A) ^1^** | **Meigs (B) ^2^** | **Wildman** |
| --- | --- | --- | --- | --- | --- |
| MHOWO | 7.6 | 14.9 | 37.7 | 53.9 | 33.7 |
| MUOWO | 70.4 | 63.2 | 40.3 | 24.1 | 44.4 |
| MHN | 3.4 | 6.5 | 17.6 | 21.0 | 15.6 |
| MUN | 18.6 | 15.4 | 4.3 | 1.0 | 6.3 |

^1^ Using metabolic syndrome variables ^2^ Using homeostasis model only. Results are expressed as the percentage of all subjects.
