# Supplementary material for: Defining Metabolically Healthy Obesity: Role of Dietary and Lifestyle Factors
Source: PLoS One. 2013 Oct 17;8(10):e76188. doi: 10.1371/journal.pone.0076188 (PMC3798285; doi:10.1371/journal.pone.0076188)
Supplement: Table S2 — Anthropometric and clinical characteristics of the Mitchelstown cohort according to metabolic health status among the normal weight and combined overweight and obese subjects. (DOCX) [file pone.0076188.s002.docx]

**Table S2.** Anthropometric and clinical characteristics of the Mitchelstown cohort according to metabolic health status among the normal weight and combined overweight and obese subject

|  |  | **Aguilar-Salinas** | *P* | **Karelis** | *P* | **Meigs (A) ^1^** | *P* | **Meigs (B) ^2^** | *P* | **Wildman** | *P* |
| --- | --- | --- | --- | --- | --- | --- | --- | --- | --- | --- | --- |
| **Age, yrs** | MHOWO | 59.6±0.4 | 0.61 | 61.1±03 | 0.000 | 58.9±0.2 | 0.000 | 59.9±0.2 | 0.96 | 59.0±0.2 | 0.000 |
|  | MUOWO | 59.8±0.2 |  | 59.6±0.2 |  | 60.8±0.2 |  | 59.9±0.2 |  | 60.6±0.2 |  |
|  | MHN | 59.4±0.6 | 0.67 | 59.6±0.5 | 0.27 | 58.4±0.3 | 0.000 | 59.1±1.4 | 0.33 | 58.2±0.3 | 0.000 |
|  | MUN | 59.1±0.3 |  | 59.0±0.3 |  | 62.4±0.6 |  | 60.3±0.3 |  | 61.7±0.5 |  |
| **BMI, kg/m^2^** | MHOWO | 29.1±0.3 | 0.001 | 29.3±0.2 | 0.000 | 28.8±0.1 | 0.000 | 29.1±0.1 | 0.000 | 28.7±0.1 | 0.000 |
|  | MUOWO | 30.3±0.1 |  | 30.4±0.1 |  | 31.5±0.2 |  | 32.5±0.2 |  | 31.3±0.2 |  |
|  | MHN | 23.0±0.2 | 0.47 | 22.8±0.1 | 0.40 | 22.8±0.1 | 0.04 | 22.9±0.1 | 0.12 | 22.9±0.1 | 0.30 |
|  | MUN | 22.9±0.1 |  | 22.9±0.1 |  | 23.2±0.2 |  | 23.5±0.3 |  | 23.0±0.1 |  |
| **Waist, cm** | MHOWO | 97.0±0.8 | 0.000 | 97.4±1.0 | 0.000 | 97.0±0.4 | 0.000 | 97.8±0.3 | 0.000 | 97.0±0.4 | 0.000 |
|  | MUOWO | 101.3±0.3 |  | 101.9±03 |  | 105.0±0.4 |  | 107.8±0.5 |  | 104.2±0.4 |  |
|  | MHN | 82.1±0.9 | 0.71 | 81.6±0.7 | 0.08 | 82.2±0.4 | 0.03 | 82.5±0.4 | 0.18 | 82.0±0.3 | 0.02 |
|  | MUN | 82.6±0.4 |  | 83.1±0.5 |  | 84.5±0.9 |  | 85.5±2.15 |  | 84.1±0.8 |  |
| **Body fat, %** | MHOWO | 36.9±0.6 | 0.26 | 38.2±0.4 | 0.047 | 36.2±0.2 | 0.000 | 36.9±0.2 | 0.000 | 36.6±0.3 | 0.000 |
|  | MUOWO | 37.6±0.2 |  | 37.3±0.4 |  | 38.7±0.2 |  | 38.9±0.3 |  | 38.2±0.3 |  |
|  | MHN | 31.8±0.7 | 0.58 | 31.9±0.5 | 0.26 | 30.9±0.3 | 0.001 | 31.4±0.3 | 0.33 | 31.2±0.3 | 0.27 |
|  | MUN | 31.4±0.3 |  | 31.2±0.3 |  | 33.3±0.6 |  | 30.1±1.34 |  | 31.9±0.5 |  |
| **TG, mmol/L** | MHOWO | 1.28±0.04 | 0.002 | 1.05±0.02 | 0.000 | 1.20±0.02 | 0.000 | 1.35±0.02 | 0.000 | 1.14±0.02 | 0.000 |
|  | MUOWO | 1.51±0.02 |  | 1.60±0.03 |  | 1.78±0.04 |  | 1.82±0.04 |  | 1.77±0.03 |  |
|  | MHN | 0.96±0.05 | 0.03 | 0.87±0.03 | 0.000 | 1.01±0.02 | 0.000 | 1.05±0.02 | 0.07 | 0.96±0.02 | 0.000 |
|  | MUN | 1.08±0.03 |  | 1.15±0.04 |  | 1.35±0.09 |  | 1.66±0.32 |  | 1.36±0.07 |  |
| **HDL-C, mmol/L** | MHOWO | 1.47±0.03 | 0.002 | 1.54±0.02 | 0.000 | 1.49±0.01 | 0.000 | 1.45±0.01 | 0.000 | 1.52±0.02 | 0.000 |
|  | MUOWO | 1.38±0.01 |  | 1.35±0.01 |  | 1.29±0.01 |  | 1.23±0.01 |  | 1.29±0.01 |  |
|  | MHN | 1.68±0.05 | 0.90 | 1.67±0.03 | 0.97 | 1.67±0.02 | 0.76 | 1.68±0.02 | 0.003 | 1.69±0.02 | 0.08 |
|  | MUN | 1.38±0.01 |  | 1.67±0.02 |  | 1.68±0.05 |  | 1.42±0.08 |  | 1.62±0.04 |  |
| **FPG, mmol/L** | MHOWO | 5.00±0.05 | 0.006 | 5.11±0.06 | 0.001 | 4.91±0.02 | 0.000 | 4.97±0.03 | 0.000 | 4.85±0.02 | 0.000 |
|  | MUOWO | 5.31±0.04 |  | 5.34±0.04 |  | 5.66±0.06 |  | 6.05±0.09 |  | 5.15±0.05 |  |
|  | MHN | 4.74±0.06 | 0.27 | 4.73±0.04 | 0.02 | 4.78±0.03 | 0.03 | 4.79±0.02 | 0.01 | 4.85±0.02 | 0.000 |
|  | MUN | 4.85±0.06 |  | 4.88±0.05 |  | 5.09±0.14 |  | 5.88±0.10 |  | 5.63±0.10 |  |
| **HOMA** | MHOWO | 2.46±0.22 | 0.005 | 2.13±0.15 | 0.000 | 2.12±0.06 | 0.000 | 1.76±0.02 | 0.000 | 1.90±0.05 | 0.000 |
|  | MUOWO | 3.32±0.10 |  | 3.54±0.10 |  | 4.34±0.15 |  | 6.64±0.22 |  | 4.29±0.14 |  |
|  | MHN | 1.35±0.11 | 0.74 | 1.19±0.06 | 0.002 | 1.29±0.05 | 0.005 | 1.22±0.03 | 0.000 | 1.20±0.04 | 0.000 |
|  | MUN | 1.39±0.06 |  | 1.48±0.07 |  | 2.12±0.06 |  | 1.76±0.02 |  | 1.90±0.05 |  |
| **SBP, mm Hg** | MHOWO | 121.9±0.9 | 0.000 | 129.2±0.9 | 0.046 | 127.1±0.7 | 0.000 | 129.6±0.5 | 0.000 | 125.6±0.6 | 0.000 |
|  | MUOWO | 131.7±0.5 |  | 131.2±0.5 |  | 134.4±0.6 |  | 133.7±0.7 |  | 134.8±0.5 |  |
|  | MHN | 118.0±1.1 | 0.000 | 126.4±1.7 | 0.40 | 122.2±0.9 | 0.000 | 125.2±0.9 | 0.21 | 120.1±0.8 | 0.000 |
|  | MUN | 126.4±1.0 |  | 124.8±0.9 |  | 137.9±1.9 |  | 130.3±3.1 |  | 138.8±1.6 |  |
| **DBP, mm Hg** | MHOWO | 76.6±0.6 | 0.000 | 78.9±0.5 | 0.000 | 79.4±0.3 | 0.000 | 80.5±0.3 | 0.001 | 78.8±0.3 | 0.000 |
|  | MUOWO | 81.5±0.3 |  | 81.5±0.3 |  | 82.4±0.3 |  | 82.3±0.5 |  | 82.6±0.4 |  |
|  | MHN | 73.7±0.7 | 0.002 | 76.4±0.8 | 0.23 | 76.0±0.5 | 0.000 | 77.1±0.5 | 0.01 | 75.1±0.5 | 0.000 |
|  | MUN | 77.6±0.5 |  | 77.6±0.5 |  | 82.2±1.0 |  | 81.8±1.6 |  | 82.4±0.8 |  |

**^1^** Values are presented as means ± SEM. ^2^ Statistical analysis conducted using Students *t*-tests. The first *p* value represents the MHOWO vs MUOWO comparison. The second *p* value represents the MHN vs MUN comparison.
