# Supplementary material for: Defining Metabolically Healthy Obesity: Role of Dietary and Lifestyle Factors
Source: PLoS One. 2013 Oct 17;8(10):e76188. doi: 10.1371/journal.pone.0076188 (PMC3798285; doi:10.1371/journal.pone.0076188)
Supplement: Table S3 — Multivariate-adjusted odds ratios for the metabolically healthy phenotype associated with demographic and lifestyle factors among the normal weight and the combined overweight and obese individuals. (DOCX) [file pone.0076188.s003.docx]

**Table S3.** Multivariate-adjusted odds ratios for the metabolically healthy phenotype associated with demographic and lifestyle factors among the normal weight and combined overweight and obese individuals

|  | **Aguilar-Salinas** |  | **Karelis** |  | **Meigs (A) ^1^** |  | **Meigs (B) ^2^** |  | **Wildman** |  |
| --- | --- | --- | --- | --- | --- | --- | --- | --- | --- | --- |
| **Gender** |  | *P* |  | *P* |  | *P* |  | *P* |  | *P* |
| Male | 1 [reference] |  | 1 [reference] |  | 1 [reference] |  | 1 [reference] |  | 1 [reference] |  |
| Female (overweight+obese) | 0.83 (0.46-1.48) | 0.52 | 2.22 (1.50-3.27) | **0.000** | 1.56 (1.15-2.14) | **0.005** | 1.80 (1.27-2.54) | **0.001** | 2.11(1.54-2.88) | **0.000** |
| Female  (normal weight) | 1.24 (0.48-3.22) | 0.66 | 1.94 (0.94-4.00) | 0.07 | 0.28 (0.11-0.72) | **0.008** | 0.79 (0.13-4.65) | 0.28 | 0.74 (0.36-1.51) | 0.41 |
| **Age group** |  |  |  |  |  |  |  |  |  |  |
| 45-54 | 1 [reference] |  | 1 [reference] |  | 1 [reference] |  | 1 [reference] |  | 1 [reference] |  |
| 55-64 (overweight+obese) | 1.25 (0.66-2.34) | 0.49 | 1.63 (1.02-2.59) | **0.04** | 0.57 (0.40-0.80) | **0.001** | 0.90 (0.62-1.30) | 0.58 | 0.61 (0.44-0.86) | **0.004** |
| 65-74 (overweight+obese) | 0.80 (0.30-2.13) | 0.65 | 3.33 (1.89-5.87) | **0.000** | 0.35 (0.22-0.56) | **0.000** | 1.16 (0.69-1.95) | 0.57 | 0.43 (0.27-0.69) | **0.001** |
| 55-64  (normal weight) | 0.90 (0.35-2.30) | 0.82 | 0.76 (0.38-1.52) | 0.44 | 0.59 (0.23-1.51) | 0.27 | 0.32 (0.04-3.70) | 0.41 | 0.43(0.19-0.98) | **0.04** |
| 65-74  (normal weight) | 0.55 (0.13-2.34) | 0.42 | 0.69 (0.26-1.85) | 0.46 | 0.13 (0.11-0.72) | **0.000** | 0.24 (0.02-3.25) | 0.28 | 0.21 (0.04-0.33) | **0.000** |
| **Dietary quality ^3^** |  |  |  |  |  |  |  |  |  |  |
| Low | 1 [reference] |  | 1 [reference] |  | 1 [reference] |  | 1 [reference] |  | 1 [reference] |  |
| High (overweight+obese) | 1.18 (0.67-2.09) | 0.56 | 1.43 (0.97-2.11) | 0.07 | 1.11 (0.82-1.52) | 0.49 | 0.92 (0.66-1.29) | 0.63 | 1.02 (0.75-1.38) | 0.92 |
| High  (normal weight) | 0.97 (0.78-1.38) | 0.56 | 0.86 (0.45-1.62) | 0.64 | 0.87 (0.41-1.82) | 0.71 | 2.05 (0.39-3.81) | 0.40 | 0.90 (0.47-1.73) | 0.75 |
| **Food pyramid compliance ^4^** |  |  |  |  |  |  |  |  |  |  |
| Low | 1 [reference] |  | 1 [reference] |  | 1 [reference] |  | 1 [reference] |  | 1 [reference] |  |
| High (overweight+obese) | 0.71 (0.40-1.25) | 0.23 | 1.17 (0.80-1.670) | 0.41 | 0.75 (0.56-1.02) | 0.06 | 0.95 (0.68-1.31) | 0.73 | 0.89 (0.66-1.21) | 0.46 |
| High  (normal weight) | 1.76 (0.74-4.20) | 0.20 | 0.76 (0.41-1.43) | 0.40 | 1.02 (0.49-2.12) | 0.96 | 0.86 (0.17-4.44) | 0.87 | 1.22 (0.64-2.33) | 0.55 |
| **Physical activity** |  |  |  |  |  |  |  |  |  |  |
| Low | 1 [reference] |  | 1 [reference] |  | 1 [reference] |  | 1 [reference] |  | 1 [reference] |  |
| Moderate + High (overweight+obese) | 0.96 (0.55-1.68) | 0.89 | 1.06 (0.72-1.55) | 0.76 | 1.20 (0.89-1.62) | 0.24 | 1.88 (1.35-2.61) | **0.000** | 1.20 (0.88-1.61) | 0.25 |
| Moderate + High  (normal weight) | 0.42 (0.18-1.00) | 0.07 | 1.84 (0.96-3.53) | 0.07 | 0.79 (0.38-1.67) | 0.54 | 1.21 (0.78-4.23) | 0.42 | 1.61 (0.84-3.05) | 0.15 |
| **Smoking** |  |  |  |  |  |  |  |  |  |  |
| Never + Former | 1 [reference] |  | 1 [reference] |  | 1 [reference] |  | 1 [reference] |  | 1 [reference] |  |
| Current (overweight+obese) | 1.49 (0.71-3.13) | 0.29 | 1.32 (0.76-2.29) | 0.32 | 0.83 (0.53-1.29) | 0.41 | 1.44 (0.86-2.42) | 0.16 | 0.76 (0.48-1.20) | 0.24 |
| Current  (normal weight) | 0.72 (0.24-2.15) | 0.55 | 0.72 (0.33-1.58) | 0.42 | 0.65 (0.28-1.52) | 0.32 | 1.52 (0.92-3.30) | 0.19 | 0.80 (0.38-1.70) | 0.56 |
| **Alcohol intake** |  |  |  |  |  |  |  |  |  |  |
| Non-drinker | 1 [reference] |  | 1 [reference] |  | 1 [reference] |  | 1 [reference] |  | 1 [reference] |  |
| Drinker (overweight+obese) | 2.39 (0.99-5.73) | 0.06 | 1.45 (0.90-2.34) | 0.13 | 1.39 (0.96-2.00) | 0.08 | 1.35 (0.91-2.00) | 0.13 | 1.38 (0.95-2.00) | 0.09 |
| Drinker  (normal weight) | 1.26 (0.74-4.20) | 0.20 | 0.82 (0.39-1.72) | 0.60 | 0.73 (0.29-1.80) | 0.49 | 1.22 (0.73-1.96) | 0.52 | 0.84 (0.38-1.86) | 0.67 |

^1^ Using metabolic syndrome variables. ^2^ Using homeostasis model only. ^3^ Dietary quality determined by DASH score. ^4^ Median food pyramid compliance score. Figures are expressed as OR (95%CI). Reference group is metabolically unhealthy within same BMI category (i.e. overweight+obese or normal weight). Each factor is adjusted for every other factor in the table.
